# Developing and validating the nursing presence scale for hospitalized patients

**DOI:** 10.1186/s12912-022-00896-0

**Published:** 2022-06-02

**Authors:** Foroozan Atashzadeh-Shoorideh, Soroor Parvizy, Meimanat Hosseini, Yosra Raziani, Fatemeh Mohammadipour

**Affiliations:** 1 Department of Psychiatric Nursing and Management, School of Nursing and Midwifery, Shahid Labbafinezhad Hospital, Shahid Beheshti University of Medical Sciences, Tehran, Iran; 2 Department of Pediatric Nursing, Nursing Care Research Center, Iran University of Medical Sciences, Tehran, Iran; 3 Department of Community Health Nursing, School of Nursing and Midwifery, Shahid Beheshti University of Medical Sciences, Tehran, Iran; 4 Department of Nursing, Komar University of Science and Technology, Sulaymaniyah, Sulaymaniyah Region Iraq; 5 Social Determinants of Health Research Center, Lorestan University of Medical Sciences, Khorramabad, Iran

**Keywords:** Design, Nurses, Scale, Nursing Management

## Abstract

**Aim:**

We developed and psychometrically tested the Nursing Presence Scale.

**Background:**

Nursing presence is a foundation for professional nursing practice; therefore, it is critical to measure this concept.

**Introduction:**

This instrument development study was designed to generate an itemized scale and psychometric testing using a sample of Iranian patients.

**Methods:**

Based on both a concept development and literature review, and finally face and content validity 44-item draft scale was generated. During November 2018–2019, 774 patients were surveyed. Exploratory and confirmatory factor analyses were used to evaluate the scale’s construct validity; concurrent and predictive reliability of the nursing presence scale were also evaluated. We also examine the weighting to scale items.

**Results:**

The analyses yielded a 36-item, 4-factor scale that adequately fit the data. Cronbach’s alpha coefficient for the whole instrument was 0.94. The intra class correlation coefficient was 0.91. Nursing Presence Scale scores were positively correlated with Revised Humane Caring Scale and predicted 25% of missed nursing care.

**Conclusion:**

This 36-item has good reliability and validity, making it useful for measuring the current condition of nursing presence.

**Implications for Nursing and Health Policy:**

Measuring the frequency of nursing presence allows for data-driven planning and upgrading the inpatient care services.

## Background

Despite the fact that health care services are expected to be based on the human values and provide attention to human welfare, it has not been very successful. Therefore, many experts consider the humanization of this system an urgent necessity [1–3]. Theoretically, philosophically, and practically, the concept of nursing presence is accepted as the most basic component of humanistic care and the underlying humanization of nursing services [4, 5].

Being with someone who needs, determines the practice of professional nursing [6] and without the presence of a nurse, health care is disintegrated [7]. Benner and Cook consider presence as one of the eight components that the nurse plays in the auxiliary role [8] and presence as a non-invasive intervention is included in the classification of nursing interventions [9, 10]. However, limited methods exist for objectifying, quantifying, and measuring this concept [5]. To maintain the integrity of nursing, there is a clear need to measure the concepts of nursing and its impact on the quality of patient care [11]. Today, health care focuses on time saving and reducing costs [12], and anything that cannot be determined and quantified seems unnecessary and non-essential [13]. On the other hand, the standardization of services and client satisfaction considered important in quality of nursing care.

Numerous studies have been identified to determine the level of patient satisfaction with various aspects of nursing care, that the highest average satisfaction with nursing care was only related to the technical-professional dimension [14, 15]. However, patients have expressed entering the world of the patient, identifying different needs, and the availability of a nurse as their most important need and all these needs are reflected in the concept of nursing presence [16, 17]. High satisfaction in the technical-professional care dimension can be due to the existence of supervision and control over this dimension by managers, its high importance from the perspective of nurses, and nurses’ skills in performing this type of tasks [18]. In addition, the small number of staff and the large number of clients may force the nurse to focus on the main tasks and abandon the tasks that are not reprimanded for [19]. While much research has focused on the biological and psychological aspects of patients, social interactions between nurses and patients have received less attention and there is a strong need to explore the social issues in nursing practice [7]. Therefore, having a tool to measure the nursing presence is necessary both to standardize the nursing services and control the nurses’ presence, and care satisfaction for each client in the health care system [20], because institutions can improve the nursing presence in staff to play a role in organizational development, patient satisfaction and increase profitability.

On the other hand, today the nursing profession faces many challenges. Technological advances threaten the caring with a humanistic approach by putting a lot of pressure on the care system, and nurses to communicate with the patient through telemedicine or other communications methods. These improvements potentially impair the nurse’s ability to be present with the patient [2]. Lack of nurses and replacing nursing staff with non-nurses are other challenges. Reducing the number of professional nurses and employing untrained nurses or other medical staff instead of specialist nurses has adverse effects on the quality of patient care [21]. Therefore, in order to maintain the discipline of nursing and prove the value of this profession for the care system, the first step is to show clearly, what the nursing profession does. This situation will be stronger when it is supported by the quantitative data [4].

According to the cases presented in the field of importance of measuring the concept of the nursing, three instruments have been officially published so far, two scales measuring the concept from the perspective of nurses [22, 23] and the other from the perspective of patients [24].

Designing a tool to measure the nursing presence from the perspective of patients seems more necessary (than measuring it from the perspective of nurses). Considering the issues such as achieving patient-centered goals, the relationship between the nursing presence with patients’ satisfaction, and that patients do not understand the technical aspects of care but can understand the presence of a nurse [7, 25].

By scoring the Presence of Nursing Scale (PONS) that measures the presence of a nurse from the patient’s point of view; based on COSMIN checklist [26]. It was found that the content validity instrument also has a high internal consistency, but the exploratory factor analysis has not been performed as a method to determine the construct validity of the instrument and the existence of multiple reverse questions is another limitation of this scale. Due to the fact that the scale is still used in limited studies [27] and its strengths and weaknesses points are not well known, so this instrument cannot be used.

To measure presence in different cultural contexts, some studies have suggested the need to develop tools that are culturally sensitive [4]. Therefore, due to the dependence of the concept of nursing presence on the culture [28] and the lack of valid instruments to measure this concept, the purpose of this study is designing and examining the psychometric properties of nursing presence scale.

## Methods

### Design

**Fig. 1:**
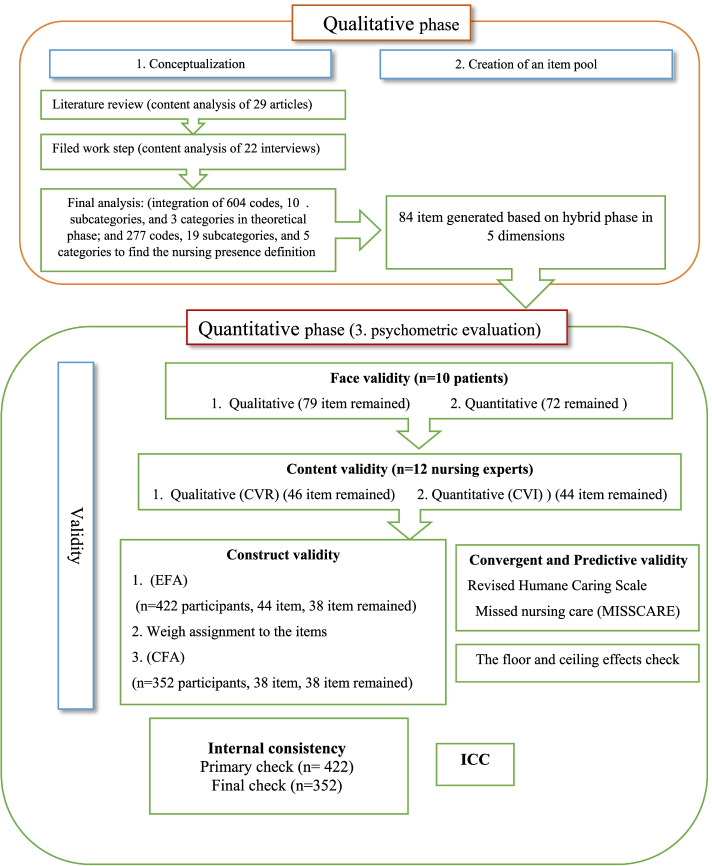
A flow chart depicting the process used to sequential exploratory design of study

Methodological study, made up of two parts: construction of a data collection instrument for the evaluation of nursing presence, and the psychometric assessment of the instrument. In this regard, three-stage exploratory mixed method (conceptualization, creation of an item pool and psychometric evaluation) recommended by Clark and Watson [29] was conducted (See Fig. 1).

#### Conceptualization

From a conceptual development [5] and empirical study [16], presence was conceptualized as a co-constructed interaction that is defined by deliberate focus, task-oriented/patient-oriented relationship, ubiquitous participation, clarification, and accountability [5]. Therefore, the dimensions of the instrument were considered according to the definition of the concept of nursing presence in the five dimensions in the level of determining content domain (Table 1).

**Table 1:** Details on levels of determining the face and content validity

Dimensions of nursing present		IS	CVR	CVI	P_**c**_	K
**Deliberate focus**	1. In the first meeting, introduce himself/herself and greets.	3.5	1	1	0.0002441	1
	2. Communicates directly with me.	3	0.16^b^			
	3. See in her/him the desire to share my problems with her/him.	5	1	1	0.0002441	1
	4. When she/he talks to me, she/he pays attention to me (and listens well)	5	0.83	1	0.0002441	1
	5. Listens well when I talk about my problems (merged with item 4).					
	6. Speaks in a calm tone while doing things	3.4	0.83	1	0.0002441	1
	7. She/he is companionable	2.7	1	1	0.0002441	1
	8. worries about me and my family	3	0.16^b^			
**Task-oriented/patient-oriented relationship**	9. Understands my feelings, emotions and worries.	3.8	1	1	0.0002441	1
	10. Tries to see things the way I see them.	1.3^a^	0.83	1	0.0002441	1
	11. Asks about things that happen in my everyday life.	2.6	− 0.2^b^			
	12. She/he is an understanding nurse	2.8	−0.83^b^			
	13. In addition to doing things, she/he also uses her/his own sense.	2.8	−0.33^b^			
	14. In addition to the results of tests and diagnostic methods, she/he also pays attention to my own experience of the disease.	3.9	1	1	0.0002441	1
	15. In addition to see the test results and the diagnostic method, she/he also uses her/his own intuition.	2	−0.2^b^			
	16. She/he understands my main problem.	2	0.33^b^			
	17. She/he understands when I need to be alone.	2	0.33^b^			
	18. In the ward, he/she call me by my name, not by the bed or room number.	2.4	1	1	0.0002441	1
	19. Patients’ concerns and conditions do not matter to her/him.	1.3	1	1	0.0002441	1
	20. In addition to physical care, she/he pays attention to my mental psychological needs.	3.5	1	1	0.0002441	1
	21. Knows each patient.	3.6	0.33^b^			
	22. She/he also asks questions about my personal life	1.6	0.0^b^			
	23. She/he accepts me as I am.	1.3^a^	1	1	0.0002441	1
	24. She is not interested in my way of thinking.	2	0.0^b^			
	25. She/he ask my opinion on matters related to my treatment.	2	0.16^b^			
	26. She/he chooses for me on my behalf.	2	0.5^b^			
	27. Asks me if I want to continue my treatment with the same doctors	3	0.16^b^			
	28. Tells me my specific information.	4.5	0.83	1	0.0002441	1
	29. Keep me informed of decisions for treatment or suggestions.	4.7	1	0.91	0.0029292	0/9
	30. I see the nurse as much as I expect.	1.2^a^	0.83	0.83	0.0161106	0.82
	31. I trust him/her.	1.6	0.83	1	0.0002441	1
	32. She/he knows all aspects of my illness.	1.7	0.83	1	0.0002441	1
	33. After identifying problems, it coordinates treatment goals with me (merged with 42).					
	34. In addition to doing things, she/he allocates enough time to myself as well.	2^a^	−0.16^b^			
	35. Pays attention to all my problems.	2^a^	−0.83^b^			
	36. She/he treats me friendly.	1.6	0.83	0.91	0.0029292	0.9
	37. She/he does not communicate with me due to her/his busy schedule.	2	0.83	^c^0.54		
**Ubiquitous participation**	38. She/he reminds me that will be with me if needed.	1.6	0.83	1	0.0002441	1
	39. I feel she/he is available whenever I need.	1.6	1	1	0.0002441	1
	40. She/he visits me regularly.	4	0.16^b^			
	41. She/he takes my worries serious.	4.7	1	1	0.0002441	1
	42. My relationship with my nurse is reciprocal.	3	0.83	0.83	0.0161106	0.82
	43. Talks to me about my care process (merged with item 42)					
	44. Treats me with courtesy and respect (and in dignity a human being).	4.7	1	1	0.0002441	1
	45. Supports the decisions I make	3	0.33^b^			
	46. She/he take a humane approach to me (merged with 44 in the level of qualitative face validity).					
	47. Knocks on the door when entering the room.	1.6	0.83	1	0.0002441	1
	48. Treats patients unfriendly.	4.7	0.0^b^			
	49. I can easily express my concerns and wishes to her/him.	4.9	0.83	0.91	0.0029292	0.9
	50. She/he can communicate with all patients with different conditions.	3.5	1	1	0.0002441	1
	51. Shares my grief.	3	0.33^b^			
	52. Behaves compassionately.	4	0.83	1	0.0002441	1
	^a^53. Reduces my fears					
	54. Encourages me to be confident.	1.7	0.83	0.83	0.0161106	0.82
	55. recounts my strengths and my conditions.	2.8	0.83	1	0.0002441	1
	56. Helps me understand my feelings.	2.5	0.16^b^			
	57. Makes available the things I need.	1.1^a^				
	58. Pays attention to the environmental conditions of the room where I am hospitalized.	1^a^				
	59. Performs his/her tasks skillfully.	5	1	1	0.0002441	1
	60. In caring, she/he also uses her/his experience in addition to having knowledge.	4.5	1	1	0.0002441	1
	61. Helps me to have the right expectations of my health.	3	0.83	1	0.0002441	1
	62. Helps me to use my previous positive experiences.	2	0.83	1	0.0002441	1
	63. There is good cooperation between the nurse and me.	2.5	0.83	0.91	0.0029292	1
	64. Offers me to choose, not to give a solution.	1.6	0.83	0.91	0.0029292	1
	65. Respects for whatever I believe.	2	0.16^b^			
	66. Provides the necessary bases for performing religious duties or rituals and festivals.	1.5	1	1	0.0002441	1
**Clarification**	67. Makes communication with the patient a priority.	3.8	1	1	0.0002441	1
	68. Explains what she wants to do for me.	4.8	1	1	0.0002441	1
	69. Teaches me some skills to be able to take care of myself.	4.9	1	1	0.0002441	1
	70. Explains the content to me in a way that is understandable.	4.7	1	1	0.0002441	1
	71. Gives me the information I want.	2	0.16			
	72. Wants me to ask any questions if I have.	3	0.83	1	0.0002441	1
	73. Answers my questions honestly.	4.7	0.83	1	0.0002441	1
	74. Explains the purpose and plans of nursing treatment.	4.6	0.83	1	0.0002441	1
	75. Asks me questions to make sure I understand.	1.3^a^	1	1	0.0002441	1
	76. Informs my companions about my condition.	2.4	0.83	1	0.0002441	1
	77. I can get help from her/him to clear up my ambiguities.	1.6	1	1	0.0002441	1
**Accountability**	78. Treats all patients equally and fairly, regardless of income level or occupation and position.	5	1	1	0.0002441	1
	79. Her/his insight is like mine.	2	0.83	^c^0.58		
	80. Is committed to her/his work and is responsible.	4.7	1	1	0.0002441	1
	81. Is committed to helping me.	3	0.16^b^			
	82. Checks that my problems have been resolved.	2	0.16^b^			
	83. Tries to solve my problems as much as possible.	3.6	0.83	1	0.0002441	1
	84. She/he leaves me alone.	2.5	0.16^b^			

^a^Eliminated in the qualitative level of face validity

^b^Eliminated in the quantitative level of face validity

^C^Eliminated in the quantitative level of content validity

#### Creation of an item pool

By extracting the phrases from text of the interviews and reviewing texts and other similar questionnaires, the initial items bank (including 100 items) was prepared based on the characteristics of the concept of nursing presence. The collected data were reviewed in several meetings with the research team under review, and similar items were merged and dimensions of the tools and items related to each dimension were finally defined based on the data, and the primary instrument was designed with 84 items.

The next step after designing an item pool is to choose the method that respondents should use. The type of answer chosen depends entirely on the nature of the question [30]. Therefore, according to the type of questions that were about occurrence rate of nursing presence characteristics, the answers should be arranged in the response and continuous package form and for this reason, the Likert type responding was chosen.

Once the type of Likert tool has been selected, the researcher must determine the number of Likert response options spectrum classes. In total, there is no standard for the number of response options on a Likert. In fact, if the number of classes in the spectrum is too large, the respondents will not be able to differentiate well between them, and if it is too low, they will not be able to report average values; as a result, the validity of the questionnaire will decrease. Therefore, an average range is desirable as far as possible [30]. In order to answer the items of this tool by patients, a 5-part Likert scale was selected (very high = 5, high = 4, medium = 3, low = 2 and very low = 1). It should be noted that at the time of determining the face validity, patients approved this form of response.

#### Psychometric evaluation

##### Face validity

Both qualitative and quantitative methods were used to determine face validity. For this purpose, the researcher interviewed 10 hospitalized patients for the quality of the basic tools, and the level of difficulty in understanding the items, the appropriateness of the items with the subject of nursing presence and the possibility of ambiguity and misunderstanding by patients were examined, and some items were removed or modified according to patients’ opinions.

In the quantitative phase, 10 patients were asked to determine the impact score of each item according to their experiences, in order to eliminate and reduce inappropriate items and if the impact score was equal to or greater than 1.5, the item was retained. A 5-option Likert continuum was considered for each item of the scale and “completely important,” “important,” “almost important,” “slightly important” and “unimportant” expressions were scored 5, 4, 3, 2 and 1, respectively. Using the item impact score formula (*Impact Score = Frequency (%) × Importance*), quantitative face validity was calculated.

After the final summation, seven items were removed yielding an instrument containing 72 items.

##### Content validity

At this level, both qualitative and quantitative methods were used to determine the content validity.

In a qualitative content validity, 12 nursing experts (including three specialists in the field of communication and nurse-patient communication; two specialists in the field of humanistic theories, two specialists in the field of making instruments, and 5 nurses experienced in clinical departments) were asked to examine the instrument in terms of the extent to which the items cover the concept and its scopes. Experts were also asked to make suggestions for modifying items of the instrument. To evaluate the content validity quantitatively, two methods of content validity ratio (CVR) and content validity index (CVI) including content validity of individual items (I-CVI index) and content validity of the whole instrument (S-CVI) were used.

For calculating CVR, expert members were asked to respond to the following options based on the Likert scale: 1 = not necessary, 2 = useful, but not essential, and 3 = essential. The CVR was approved based on the following formula:

$$CVR=\frac{the\ number\ of\ specialists\ who\ have\ checked\ option\ 3-\left(\ the\ total\ number\ of\ specialists/2\right)}{the\ total\ number\ of\ specialists/2}$$

Based on the recommendation of Lawshe [31], items whose CVR was higher than 0.56 were preserved.

To check the CVI, the respondents were asked to determine the degree of relevance of each item in the instrument from a score of 1 to 4 (1 = not relevant, 2 = somewhat relevant, 3 = highly relevant, and 4 = quite relevant). The content validity index for each item (I-CVI index) was then calculated based on the following formula:

$$ICVI=\frac{the\ Number\ of\ the\ specialists\ who\ have\ checked\ option\ 3\ and\ 4\ }{the\ total\ number\ of\ specialists}$$

In newly designed instruments, reaching an agreement of 80% or more is recommended. According to this index, items with a score higher than 0.79 are suitable, between 0.7 and 0.79 need to be corrected, and less than 0.70 are unacceptable [32].

To calculate the I-CVI, Polit proposed the Modified Kappa (K*) statistic, which is an indicator of agreement between evaluators that provides instrument designers with the information on the degree of agreement without a chance share. Chance agreement can occur in examining the indicators of agreement between evaluators, especially when the four-point scoring is placed in two related and unrelated categories. In the present study, the K* was calculated based on the following formula:

$$PC=\left[\frac{N!}{A!\left(A-N\right)!}\right]\times {0.5}^N\kern1em K\ast \frac{\left(1- CVI\right)-(PC)}{1-(PC)}$$

N = number of agreements related to relevancy; A = number of evaluators.

Modified kappa with the cutting points between 0.4 to 0.59 is considered weak, between 0.6 to 0.74 is good and more than 0.74 is excellent [33].

To evaluate the S-CVI, two approaches including Scale-level Content Validity Index/Universal Agreement Calculation Method (S-CVI/UA) and Scale-level Content Validity Index/Averaging Calculation Method(S-CVI/Ave) were calculated.

First, in the universal agreement approach, after merging the answers of “quite relevant”, and “highly relevant “ together and merging the answers of “not related” and “somewhat related” together, dual mode options of related and unrelated are formed for each expression. Then the number of items recognized by all the experts (the number of items with a content validity index = 1) is divided by the total number of items.

In the average approach, the sum of the ICVI is divided by the total number of items. S-CVI / Ave with cutting points between 0.8 to 0.9 and more than 0.9 are recognized good and excellent, respectively, and if S-CVI / UA is higher than 90%, it will be accepted [32].

##### Construct validity (factor analysis)

**Exploratory factor analysis (EFA)**

In this study, exploratory factor analysis based on the steps proposed by Nunnally & Bernstein was used to investigate the factor structure of the nursing presence instrument. Kaiser-Meyer-Olkin (KMO) test for sampling adequacy for factor analysis and the Bartlett’s Test of Sphericity for correlation between data were performed before the factor analysis [34].

Before factor analysis, Cronbach’s alpha coefficient of the questionnaires was also calculated. Then the correlation matrix of the variables was examined. Variables with a correlation below 0.3 should be omitted, as they indicate a lack of a correlation pattern, and variables with a correlation coefficient above 0.9 indicate a multi co-linearity and should be omitted [35].

In the first level of EFA, with maximum likelihood by the use of oblique rotation of the Promax type was utilised to determine the degree to which the developed instrument measures the concept of nursing presence. The criteria used for deleting items were as follows: factors that have less than three variables, or a large number of complex variables that have weigh less than 0.5 and items loaded onto more than one factor with similar loadings [36]. In the present study, eigenvalues and Scree Plot diagram were used to extract the factors of nursing presence instrument.

**Confirmatory factor analysis (CFA)**

To verify the fit of the factor structure derived from the EFA, confirmatory factor analysis was performed in another sample. To assess the goodness of fit of the data, some indices used included the χ2/degree of freedom (χ2/df), goodness-of-fit index (GFI), comparative fit index (CFI), Tacker-Lewis’s index (TLI), adjusted goodness-of-fit index (AGFI), and root mean square error of approximation (RMSEA). For the χ2/df, a value < 3 and > 1 was considered sufficiently acceptable. For the CFI, GFI, AGFI and TLI, values higher than 0.9, 0.8–0.89 and 0.7–0.79 reveal “excellent,” “good” and “acceptable” fit, respectively, while RMSEA < 0.08 reflecting an acceptable model [37].

##### Convergent and predictive validity

To verify the convergent validity of the nursing presence instrument, its correlation efficiency with the Persian version of Revised Humane Caring Scale (RHCS) was calculated. The RHCS includes 46 items categorized under the following five subscales: maintenance of social relations and privacy; communication and participations; respecting patients’ feelings; maintaining and promoting physical health; and ensuring the necessary conditions for humane caring on the ward. The Cronbach’s alpha value for these five subscales range from 0.77 to 0.96 [38]. Persian translation and psychometric evaluation of RHCS has been done by Jafaripour et.al [39].

To verify the predictive validity of the nursing presence instrument, its correlation efficiency with the Persian version of MISSCARE was calculated. The original MISSCARE was developed to measure of missed nursing care. This instrument includes 24 items across four subscales: Assessment; basic care interventions; individual care interventions; and planning that asks nurses to rate the frequency of elements of nursing care that are missed by them on their unit. Higher scores reflecting perception of more missed care. The reliability has been established by test– retest evaluation (r = .87) [40]. Persian translation and psychometric evaluation of MISSCARE has been done by Khajooee et.al [41].

### Participants

Purposeful sampling was used for EFA and CFA from selected public and private hospitals in Iran from January 2018–April 2019. First, the country was divided into five parts. Then, the share of each province was determined and according to the ratio of public and private hospitals in each province, the samples were divided, and then, some wards were selected from each hospital, and after each ward, all patients who met the inclusion criteria completed the instruments. The sample size for EFA and CFA was calculated to be 5–10 times as many subjects as the number of scale items [42] considering a 20% sample loss rate. Therefore, the required sample content calculation formula is as follows: sample size = 44 Item × (5–10) times × (1 + 20%) =264–528 cases. The number of samples per item was estimated to be eight. In the EFA stage according to the 44 remaining items of the instrument, 422 people and in the CFA stage according to the 36 remaining items; 352 patients were counted.

Inclusion criteria were: at least 18 years old, ability to participate in the study in terms of physical condition (being conscious), ability to read, write, speak and understand Persian. The other criteria were: having consent to participate in the study, at least 2 days have passed since their hospitalization and the ability to distinguish between nurses and other health care providers (according to hospital regulations based on the type of clothing or etiquette of the nurse). Exclusion criteria included unwillingness or inability to continue research.

### Reliability

#### Internal consistency

In the present study, calculation of Cronbach’s alpha of each dimension separately, and overall Cronbach’s alpha of the instrument were used in three levels, one after content validity (experimentally by 30 patients) and the other before and after factor analysis (by 422 people) were used, to determine the internal consistency.

#### Stability

In this study, stability assessment was performed through test-retest. The instrument was given to 30 patients and was completed again 2 weeks later. The scores obtained in this level were calculated using the Intra-Class Correlation Coefficient (ICC) test between answering the questionnaire twice. This test is the most acceptable statistical test for calculating stability. For ICC, values < 0.50 indicate “poor reliability”; values between 0.50–0.75 indicate “moderate,” values between 0.75–0.9 indicate “good reliability,” and values greater than 0.90 indicate “excellent reliability” [43].

The floor and ceiling effects refers to when the patients’ scores are at the highest and lowest scores range. If the ceiling and floor effect is observed, it is as if a large amount of data is not usable at the beginning and end of the table. As a result, people with the highest and lowest possible scores are not distinguishable from each other, and therefore reliability is reduced [26]. In the current study to determine the ceiling and floor effect, if 15% of people obtained a score above 80 % or a score below 20 %, existence of the ceiling and floor effect is indicated.

#### Weight assignment to items

Before conducting CFA In order to rank the items, the weight of each item was identified and determined. Considering equal weight for items of one scale reduces the external validity and generalizability of the results. Therefore, for adaptation of the research plan to the existing facts of the sample group and to avoid predetermined weights of the Likert s, using the weight assignment methods is recommended.

In other words, the weight assignment to items and variables makes it possible to estimate the presented statistical model regarding the relations of external world phenomena with high accuracy and reliability [36].

After the factor loads of each item were determined in EFA, the factor loads of each item were multiplied by the total variance value explained for the factor on which the question was based, then, the ratio of each secondary values to the sum of the secondary values was calculated to find the weight of each item in total. The result of this process was the weight assignment of each item throughout the whole instrument. After determining the weight of each item based on the factor analysis, the average weight of each item was calculated according to both methods, i.e., fixed weights equal to one for all items and weight assignment to items using the factor analysis. In the first method, the work process was such that the weight of the items was assumed to be equal to 1, then using Friedman test, the average weight of Likert values in each item of the nursing presence scale was calculated. In the second method, after determining the weight of each item, the weights obtained in factor analysis of each item were firstly multiplied by the Likert values of the response items and then, the mean rank of weighted Likert values in each item was calculated using Friedman test. Paired t-test was used to compare the mean weights obtained using both methods.

#### Data analysis

In the instrument psychometrics level; explanatory and confirmatory factor analysis, internal consistency, statistical weight of items and descriptive statistics of data were performed using SPSS software version 21 and AMOS 24.

## Results

The Nursing presence scale with 84 items was prepared at first as a draft and prepared for the psychometric process. According to the Table 1, this instrument had five dimensions, which were deliberate focus (8 items), task-oriented/patient-oriented relationship (29 items), ubiquitous participation (29 items), clarification (11 items) and accountability (7 items).

### Face and content validity

In the level of determining the face validity of the instrument, according to the patients’ opinion, 3 sets of items were merged because their content was viewed as overlapping (items 4 and 5, 44 and 46, and finally 33 and 43, overlapped with item 42), therefore they were merged.

Also, in the case of item 53 “It reduces my fears”, all the patients expressed that there is no real fear at the time of hospitalization and there is concern more because item 41 was about reducing anxiety, so item 53 was deleted. (See Table 1).

In the level of determining the quantitative face validity, 7 items with IS less than 1.5 were removed and draft instruments with 72 items entered the content validity level.

In determining the content validity by qualitative method, some items were slightly modified based on the feedback received.

In the level of determining the content validity by quantitative method, 26 items with a cut point less than 0.667 were deleted in determining the CVR. then I-CVI index was firstly calculated for the remaining 46 items, that the scores of all items except two items were higher than 0.79, and two items with a score of 0.58 and 0.54 were removed (items 37 and 79 based on Table 1).

In determining S-CVI, the score of 0.82 was obtained by the mean approach, it was interpreted good, and with the general agreement approach (U-CVI), the elementary instrument scored 0.94 that was considered excellent. The modified kappa for the items was higher than 0.82, therefore it is excellent in this respect (see Table 1).

### Exploratory factor analysis

The primary instrument entered the construct validity level with 44 questions.

In order to perform the EFA, the designed instrument was given to 422 informed patients hospitalized after obtaining the written consent. Of this, 417 questionnaires were completely completed. The average length of hospital stay was 6.9 ± 2.9 and ranged from 2 to 15 days; the history of the disease was averagely 3.8 ± 4.7 and ranged from 0 to 22 years. 60.6% were hospitalized in internal wards, 35.2% in surgical wards and 4.2% in intensive care and emergency wards. Of these, 79.2% were hospitalized in public hospitals; 6.9% were in the Social Security Hospital and 11.2% in the private hospital and other hospitals.

According to KMO test = 0.933, the number of samples was large enough to perform the factor analysis, and the significance of the Bartlett’s Test of Sphericity (approx. Chi-Square = 22,549.25, df = 946, *p* < 0.001) indicates that there are suitable conditions for performing the factor analysis. In addition, before performing factor analysis, Cronbach’s alpha coefficient of 417 questionnaires was calculated which was 0.937. Also, the analysis of items to examine the correlation above 0.9 and below 0.3 showed that the correlation coefficient of all items is in the range of 0.3–0.9.

**Fig. 2:**
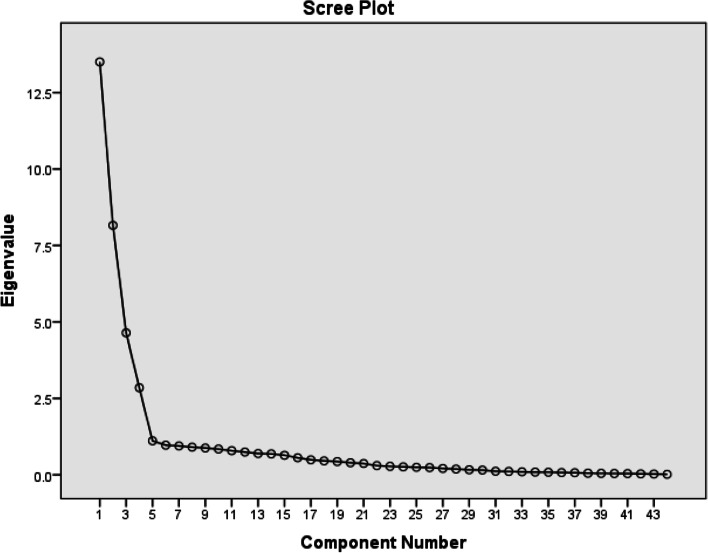
Scree plot of the nursing presence scale

According to the total variance table explained, there are five factors with a specific eigenvalue above 1, which indicates that about 68.77% of cumulative variance is explained by the first 5 factors, and 31.23% of cumulative variance by 39 other factors. The Scree Plot diagram also shows that five factors explain the factor structures of the instrument (See Fig. 2).

To determine the correlation between the variables, the factors transformation matrix was used and the minimum factor load was considered 0.5. In this level, eight items had a factor load of less than 0.5, which were removed according to opinion of the research team. It should be noted that the fifth factor in the matrix presented in the table had an item with a factor load of 0.47, which was removed. Factor analysis was performed again with four factors, and this item was still in the second factor with a factor load of 0.42, which was removed (See Table 2). Then four other factors were named. After extracting the factors and items in each factor, the degree of compatibility of the factors with the main concept and its dimensions was examined and only item three “when she talks to me, she pays attention to me and listens well” was in the third factor. Thus, it was transfer to second factor due to its greater connection with the items of this factor.

**Table 2:** Rotated Component Matrix

Item Labels	Component				
	1	2	3	4	5
41. Helping to clear up ambiguities	.942				
35. Teaching skills	.933				
34. Explain about things	.930				
28. Right expectation of the health	.917				
25. Retell the positive points	.910				
6. Understand feelings and emotions	.899				
36. Comprehensibility of the description	.879				
24. Encourage self-confidence	.861				
7. Pay attention to one’s experience	.851				
33. Prioritize communication	.842				
38. Answer honestly	.832				
9. Pay attention to the uniqueness	.830				
26. Perform tasks with skill	.782				
27. Having knowledge and experience together	.705				
37. Possibility to ask questions	.494				
1. Introduce him/herself	.487				
29. Using experiences	.479				
19. Respectful encounter		.956			
42. Treat all patients equally		.950			
23. Compassionate behavior		.949			
5. Being affable		.939			
14. Being friendly		.923			
8. Calling by last name		.900			
15. Presence if needed		.864			
21. Freedom to express concern		.786			
22. Paying attention to psychological needs		.733			
2. Willingness to communicate		.692			
4. Calm tone		.386			
31. Suggestion, not choose a solution		.313			
44. Try to fix problems			.918		
43. Commitment			.909		
32. Provide context of...			.905		
13. Know all aspects			.878		
17. Take worries seriously			.875		
3. Attention and listening			.761		
16. Feeling of availability			.712		
18. Two-way communication				.873	
11. Being in a decision				.847	
10. Giving personal information				.833	
39. Explanation of purposes and treatments				.707	
30. Good cooperation with the nurse				.639	
12. Having confidence in the nurse				.438	
40. Inform companions				.350	
20. To knock on door		.425			.477

The total number of items reached 36. The first factor, explaining 26.11% of the total variance and including 14 items, was named participation and assistance. The second factor, explaining 19.16% of the total variance, including 11 items was named conscious focus and receptive encounter. The third factor, explaining 12.46% of the total variance and with 7 items was named as monitoring and accountability, and the fourth factor, explaining 8.45% of the total variance and containing 5 items was named as coordination in care (see Table 3).

**Table 3:** Nursing presence scale (final version) and Item weight

Dimens	Items	1	2	3	4	Secondary values	***Item weight***
		The ratio of the variance of each factor to the total variance					
		20.398	12.322	7.015	4.300		
**Participation and assistance**	1. I can get help from her/him to clear up my ambiguities.	943/0				19.235	4.664
	2. Teaches me some skills to be able to take care of myself.	933/0				19.031	4.615
	3. Explains what she wants to do for me.	0.930				18.970	4.600
	4. Helps me to have the right expectations of my health	0.917				18.704	4.536
	5. Recounts my strengths and my conditions.	0.910				18.562	4.501
	6. Understands my feelings, emotions and worries	0.899				18.337	4.447
	7. Explains the content to me in a way that is understandable	0.879				17.929	4.348
	8. Encourages me to be confident on myself	0.861				17.652	4.280
	9. In addition to the results of tests and diagnostic methods, she/he also pays attention to my own experience of the disease	0.851				17.358	4.209
	10. Makes communication with the patient a priority.	0.842				17.175	4.165
	11. Answers my questions honestly	0.832				16.971	4.115
	12. In addition to physical care, she/he pays attention to my mental psychological needs.	0.830				16.930	4.105
	13. Performs his/her tasks skillfully.	0.782				15.951	3.868
	14. In caring, she/he also uses her/his experience in addition to having knowledge.	0.705				14.380	3.487
**Conscious focus and receptive encounter**	15. Treats me with courtesy and respect (and in dignity a human being).		0.956			11.779	2.856
	16. When she/he talks to me, she/he pays attention to me (and listens well)		0.950			11.705	2.838
	17. treats all patients equally and fairly, regardless of income level or occupation and position		0.949			11.693	2.835
	18. Behaves compassionately		0.939			11.570	2.805
	19. She/he is affable		0.923			11.373	2.758
	20. She/he treats me friendly.		0.900			11.089	2.689
	21. In the ward, he/she call me by my name, not by the bed or room number.		0.864			10.646	2.851
	22. She/he reminds me that will be with me if needed.		0.786			9.685	2.348
	23. I can easily express my concerns and wishes to her/him.		0.733			9.032	2.190
	24. She/he can communicate with all patients with different conditions		0.692			8.526	2.067
	25. See in her/him the desire to share my problems with her/him		0.956			6.439	1.561
**Monitoring and accountability**	26. Tries to solve my problems as much as possible			0.918		6.376	1.546
	27. Is committed to her/his work and is responsible.			0.909		6.348	1.539
	28. Provides the necessary bases for performing religious duties or rituals and festivals.			0.905		6.159	1.493
	29. She/he knows all aspects of my illness			0.878		6.138	1.488
	30. She/he takes my worries serious			0.875		5.338	1.294
	31. I feel she/he is available whenever I need			0.761		4.994	1.211
**Coordination in care**	32. My relationship with my nurse is reciprocal.				0.873	3.753	0.910
	33. Keep me informed of decisions for treatment or suggestions.				0.847	3.642	0.883
	34. Tells me my specific information				0.833	3.581	0.868
	35. Explains the purpose and future plans.				0.707	3.040	0.837
	36. There is good cooperation between the nurse and me.				0.639	2.247	0.544
**Total secondary values**						412.338	

### Weigh assignment to the items

After determining the factor load of each item, it was found that the factor load of all 36 items in total is greater than 0.4 and is suitable for ranking. Then the weight of each item was calculated. Comparing the average weights obtained using both paired t-test methods showed that there is a significant difference between the two methods [36]. Therefore, to better interpret the findings of the scale, it is better to calculate the weighted Likert values. Accordingly, at the end, the weight of the items was rounded and the range of scores was calculated. The total score of the instrument is in the range of 95–475, with values of 95–220 indicating the low presence, 221–349 the moderate presence and 350–475 the high presence (see Tables 3 and 4).

**Table 4:** Calculation of scores of nursing presence items (answer key)

factor	items	weight	Range of item scores	Range of factor scores
Participation and assistance	1–14	4	4–20	56–280
Conscious focus and receptive encounter	15–25	2.5	2.5–12.5	27.5–137.5
Monitoring and accountability	26–31	1.5	1.5–7.5	9–45
Coordination in care	32–36	0.5	0.5–2.5	2.5–12.5

### Reliability

In the first level, after determining the content and face validity of the primary instrument, the internal consistency of the initial scale was determined as a pilot in a sample of 30 patients eligible to participate in the study. Cronbach’s alpha of the whole scale was 0.91. In the second level, before performing the factor analysis, a sample of 437 eligible patients was calculated in which the Cronbach’s alpha was 0.937. After performing factor analysis and deleting items, the overall alpha of the instrument was 0.94.

In the next step, ICC was calculated with the model (Two-Way Mixed) with the confidence of 0.95 and relative stability, which was equal to 0.91(see Table 5). In the study of the ceiling and floor effect, 51 people (11.6%) had a score higher than 176 and 8 people (1.83%) had a score lower than 88 and according to the results of percentages, the effect of ceiling and floor was not observed.

**Table 5:** ICC coefficient of the instrument after factor analysis

factor	Intra class correlation	Mean ± SD	95% confidence interval	Error variance	*p* value	Interpretation
1	0.90	95.23 ± 10.10	88.85–101.61	3.19	< 0.001	Excellent
2	0.90	83.66 ± 12.43	75.80–91.52	3.93	< 0.001	Excellent
3	0.86	47.73 ± 5.24	43.81–51.66	1.96	< 0.001	Good
4	0.81	39.23 ± 6.68	33.41–45.05	2.91	< 0.001	Good
TOTAL	0.91	28.70 ± 32.04	261.48–299.92	9.61	< 0.001	Excellent

### Confirmatory factor analysis

**Fig. 3:**
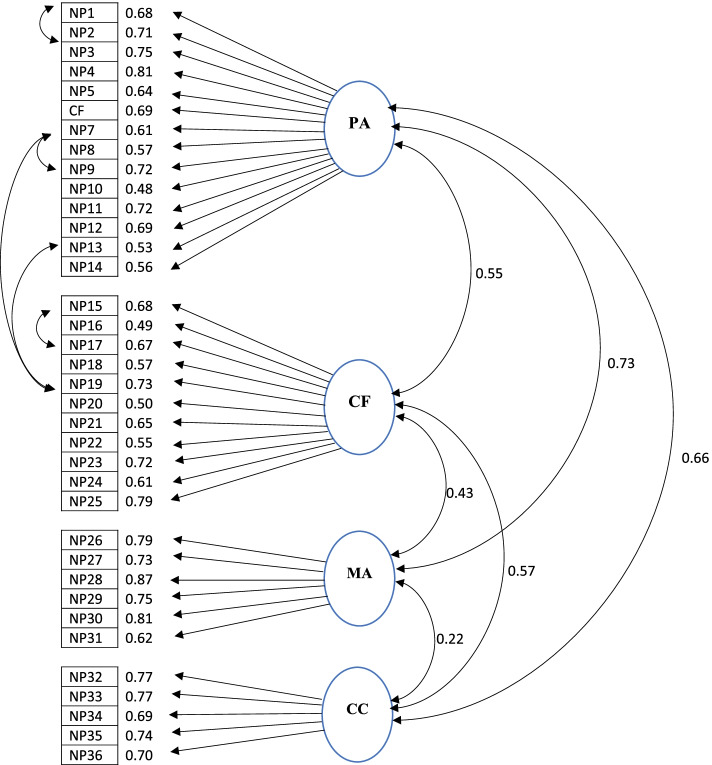
Model fitness. PA: (Participation and assistance); CF: (Conscious focus and receptive encounter); MA: (Monitoring and accountability); CC (Coordination in care)

Before the CFA, the normality of the data was measured using the Kolmogorov-Smirnov test and the Q-Q plot diagram, and since the data distribution was normal, structural equation modeling (SEM) was used. In CFA, four conceptual factors were components of the model of nursing presence scale (See Fig. 3). Goodness-of-fit criteria were calculated, which demonstrated a good fit of the model as follows: χ2/df = 2.75, RMSEA = 0.069, CFI = 0.903, and TLI = 0.896.

### Convergent and predictive validity

The Persian version of the RHCS was positively correlated with the nursing presence scale (r = 0.74, *p* < 0.001), indicating that these two scales measured constructs that are theoretically related. In contrast, the Persian version of the MISSCARE had a negative relationship with the nursing presence scale (r = − 0.47, *p* < 0.001), indicating that the nursing presence can be a factor in reducing missed nursing care.

## Discussion

The primary instrument was designed in 5 dimensions and 84 items, based on the findings of the concept development and literature review level.

In the quantitative level of the present study, which was conducted with the aim of designing and psychometrics of nursing presence instrument from the perspective of Iranian patients, the nursing presence scale was finalized with 36 phrases in four dimensions.

The participation and assistance dimension represents the nurse assistance to the patient to discover the power of healing within themselves by facilitating their self-knowledge, by determining their life patterns. This dimension does not mean the nurse’s responsibility in recovery of patient from illness to health or disease prevention. Nurse in presence uses herself/himself as a therapeutic tool to help patients identify how their living conditions and decisions may affect their recovery capacity.

The monitoring and accountability dimension indicates the continuous, conscious monitoring of the nurse and the attention and following up the care provided to meet the patient’s physical and mental needs.

Dimension of focus on the patient and the receptive behavior includes the conscious focus on the patient, friendly behaviors, and affability, considering the uniqueness of each patient and in general, the unconditional acceptance of the patient, including the human dignity.

Dimension of coordination in care reflects the patient’s authority in her/his health status and the presence of the nurse as a facilitator and companion of the patient in understanding treatment options, their effects and choosing interventions.

In the present study, internal consistency was calculated in two levels of whole scale and scale dimensions and these statistics showed nursing presence scale has a good internal consistency.

In PONS, internal consistency of the whole scale with the above method is reported to be 0.95 [24].

The stability of the present scale determined by the ICC. Also in the present study, the distribution of responses through the effects of floor and ceiling was investigated, and this effect was not observed. In this way, reliability of the present instrument stability is excellently interpreted. Stability on the scale presented by Kostovich is reported to be 0.79 with Spearman correlation coefficient, measurement error and intra-class agreement have not been investigated using ICC, while in determining the reliability of an instrument, the stability test is required according to the COSMIN checklist [44].

Nunnally and Bernstein state that for the basic decisions based on an instrument, its minimum alpha should be 0.9. According to the alpha coefficient of 0.94 obtained by this scale, this instrument can be used for basic decisions such as the nurse-to-patient ratio or staff categories composition of the ward.

As mentioned so far, only a valid scale has been introduced to measure the presence of nursing from the patients’ point of view. According to these questions, we can point out some points about the similarities and differences between this instrument and the instrument designed in the current study:

In designing items for an instrument, the purpose of the instrument design should be considered. If the goal is to measure a concept, the questions should examine a single concept and its characteristics and distinguish it from similar concepts [30]. Given that the scale designed by Kostovich [24] was designed to measure the patient’s experience of nursing presence; so, questions such as “did these nurses make me feel safe” and “did these nurses improve my quality of life” and or “did I trust these nurses” measure the consequences of a nursing presence and seems unnecessary.

Promoting shared decision making is one of the main areas that has been considered in the presence of nursing as part of holistic care. Mariano considers this process as engagement versus compliance, and it is believed that patients have an inner capacity for self-healing and self-direction [45]. Nurses must respect and support this right and capacity. At the scale designed in the present study, this case has been well considered in terms of coordination in care, and it seems that failure to pay attention to this important item is of the PONS constraints.

Other limitations of PONS include inverse questions. Polit and Beck state that although in the past it was assumed that the presence of reverse questions in the questionnaire could help reduce the chance response, today it has been found that the presence of reverse questions make respondents confused by the questionnaire [46]. In the instrument designed in the present study, some inverse questions were firstly provided in the question bank, but they were removed according to the opinion of experts in the next level, and finally, a 36-item psychometric instrument was presented in a positive way.

The four factors of the final instrument differ slightly from the five initial factors originally intended for the instrument. Thinking on the data and results shows that these four dimensions are empirically related to the initial dimensions. For example, out of 22 total items related to the two dimensions of comprehensive participation and clarification in the primary instrument, 11 items are included in participation and assistance dimension of the final instrument. All items of deliberate focus are in the dimension of conscious focus and receptive encounter. The items of task-oriented/patient-oriented relationship in the primary instrument is also distributed more in dimensions of conscious focus and receptive encounter, participation and assistance, and coordination in care. It should also be noted that in the real world, experiences for individuals are as holistic and integrated and in the qualitative level, we are forced to separate and classify the findings for discussion and presentation. Therefore, this slight difference may be observed because the findings in the qualitative level were extracted from less individuals in a different way, and are collected from more people in a different way in the quantitative level. The final dimensions of the instrument show a more logical description of the nursing presence in practice. Through the presence of nursing as a cohesive interaction, nurses around the world can work with patients to achieve their ultimate goals.

### Implications for Nursing and Health Policy

Measuring the frequency of nursing presence allows for data-driven planning and upgrading the inpatient care services. Therefore, the designed instrument can be used to design the experimental indicators such as evaluation tools and intervention protocol. Due to the importance of measuring concepts with the valid and reliable tools in research, and the time-consuming and costly nature of psychometric research, so the designed instrument can be facilitating for research related to the presence of nursing. The presence of nursing can lead to patient satisfaction and healing, and more studies need to be designed to validate these results with instrument designed in the multiple quantitative studies.

The Nursing presence scale can also play a vital role in research related to nursing presence and its related factors, assessing of the current situation, reviewing the presence process at different times and places and conditions. It can use for measuring and applying the results of interventions in this field, improving and maintaining the executive quality of nursing cares (including calculating the ratio of nurse to patient and calculating the ratio of different categories of nurses in each ward) and evidence-based care.

## Conclusion

Since the human aspects of care are important due to the cultural and religious considerations, international, cross-cultural research on the presence of nursing is recommended. Designing an instrument to measure the presence of nursing from the patients’ point of view showed the acceptable degrees of validity and reliability.

## Data Availability

The data sets used and analyzed during this study can be provided from the corresponding author on reasonable request.
